# 

**Published:** 1850-04

**Authors:** 


### CONTENTS

OF THE

### MEDICAL EXAMINER.

NEW SERIES—NO. LXIV.—APRIL, 1850.

### ORIGINAL COMMUNICATIONS.

On the proportion of Graduates to the Population. By George Tucker, Esq., formerly Professor of Moral Philosophy in the University of Virginia, - - - - - - - - - - - - 199

Contributions to Anatomy and Physiology. By W. R. Handy, M. D., Baltimore, - - - - - - - - - - - - 202

Case of Emphysema of the Sub-Mucous Cellular Coat of the Stomach; with remarks. By Francis West, M. D., of Philadelphia, - 205

Philadelphia County Medical Society.—Oil of Turpentine in Typhus Fever, etc., - - - - - - - - - 208

### BIBLIOGRAPHICAL NOTICES.

A Universal Formulary, containing the methods of preparing and administering officinal and other medicines; the whole adapted to physicians and pharmaceutists. By R. Eglesfeld Griffith, M. D., - - - - - - - - - - 220

Much improved and extended. The Medical Formulary, being a collection of prescriptions derived from the writings and practice of many of the most eminent physicians in America and Europe; to which is added an Appendix, containing the usual dietetic preparations and antidotes for poisons: the whole accompanied with a few brief pharmaceutical and medical observations. By Benjamin Ellis, M. D., &c., - - - - 220

The History of the Cholera in Exeter in 1832. By Thomas Shapter, M. D., Physician to the Devon and Exeter Hospital, &c., - 226

Transactions of the American Medical Association.—Report on Medical Education, - - - - - - - - - - - - - 230

Half-Yearly Abstract of the Medical Sciences. By Dr. Ranking, - 238

Report of the Pennsylvania Hospital for the Insane, for the year 1849. By Thomas S. Kirkbride, M. D., Physician to the Institution, - 239

Charge to the Graduates of Jefferson Medical College of Philadelphia, delivered at the public commencement, held March 9th, 1850. By J. K. Mitchell, M. D., - - - - - - - - - - 240

### EDITORIAL.

Choice Extract, - - - - - - - - - - - - - 242

Introductory Lectures, - - - - - - - - - - - - 245

Students and Graduates, - - - - - - - - - - - 245

Death of Marcus L. Taft, M. D., - - - - - - - - - - 246

Medical Practitioner's and Student's Library, - - - - - 246

Delegates to the Convention for Revising the National Pharmacopœia, 246

Medical Society of the State of Pennsylvania, - - - - - - 247

Summer Medical Schools, - - - - - - - - - - . 247

University of New York—Medical Department, - - - - - 247

Army Medical Board, - - - - - - - - - - - - - 248

A Gentle Hint to Non-Subscribing Correspondents, - - - - 248

Ovariotomy, - - - - - - - - - - - - - - 248

Deaths in Philadelphia during the months of January, February and March, (up to March 23d.) Reported by Mr. James Aitken Meigs, Student of Medicine, - - - - - - - - 249

### RECORD OF MEDICAL SCIENCE.

### PATHOLOGY AND PRACTICE OF MEDICINE.

Treatment of Dysentery by Injections of Nitrate of Silver and Creosote. By Professor Flint, - - - - - - - - 252
Neuralgia and Rheumatism treated by cold draughts after sweating, 253

### SURGERY.

New Method of Operating for Extirpation of the Testicle, - - 254
Prevention of the entrance of Air when removing Fluid from the Pleuræ, Peritoneum, and Cavities of Abscesses, - - - 254

### OBSTETRICS.

Effects of the Moral Treatment of Hysterical Fits, - - - - - 255
Obesity Simulating Pregnancy—Caution in the Diagnosis of Pregnancy. By Dr. Leopold Schonburgh, - - - - - - 255
Influence of Cholera upon Pregnancy and the Fœtus, - - - 256

